# Reviewed article: Lopes MS, Silva VMSM, Ferreira NL, Carvalho MCRC, Freitas PP, Lopes ACS. Adherence to dietary practices recommended by the Dietary Guidelines for the Brazilian Population among people with obesity: baseline of a community trial carried out at the Health Fitness Program in Belo Horizonte, state of Minas Gerais, Brazil, 2022-2023. Epidemiol Serv Saude. 2025:34;e20240287

**DOI:** 10.1590/S2237-96222025v34e20240287.a

**Published:** 2025-04-28

**Authors:** Tatiana Sadalla Collese

**Affiliations:** Universidade de São Paulo (USP)

**Table:** 

Rating	Excellent	Good	Average	Below average	Poor
Interest		✓			
Quality		✓			
Originality		✓			
Overall		✓			

**Table:** 

Questionnaire	Yes	No	Not applicable
Does the manuscript contain new and significant information to justify publication?	✓		
Does the Abstract (Summary) clearly and accurately describe the content of the article?		✓	
Is the problem significant and concisely stated?	✓		
Are the methods described comprehensively?	✓		
Are the interpretations and conclusions justified by the results?	✓		
Is adequate reference made to other work in the field?	✓		

## Manuscript Structure:

Length of article is: Adequate

Number of tables is: Adequate

Number of figures is: Adequate

Please state any conflict(s) of interest that you have in relation to the review of this paper (state “none” if this is not applicable): None

## Comment

Prezados autores,

O manuscrito é relevante e atual.

Abaixo estão algumas considerações:

- Resumo: Nos resultados deixar apenas o IC95%, sem a necessidade de valor de p. O último OR não está claro a qual variável este se refere. A conclusão está muito generalizada, favor reescrever de acordo aos principais achados do estudo.
- Introdução: Está clara e bem delineada.
- Limitações: Apesar dos autores terem citado o Programa Academia da Saúde nas limitações do estudo, é importante acrescentar que o fato dos participantes fazerem parte deste programa faz com que eles recebam orientações alimentares feitas por nutricionistas ou outros profissionais da atenção básica, que tendenciem os resultados referentes as adesões propostas pelo Guia Alimentar. Isso pode representar viés de seleção e de informação.
- Outro importante viés é que a amostra é majoritariamente feminina.
- Métodos: Quando aconteceu a triagem e a linha de base? Incluir meses e ano.
- Em que momento o peso e a altura foram aferidos?
- Discussão: A figura 1 traz resultados importantes, mas que não foram discutidos no texto. Por exemplo, fazer as refeições à mesa; participar do preparo de alimentos; variar o consumo de feijão e comer fruta no café da manhã.
- Estes podem ser melhor relacionados com os resultados da figura 3.
- Conclusão: O último parágrafo traz as principais fortalezas do estudo, porém não responde claramente ao objetivo do estudo. Favor reescrever.

